# The *eLife* approach to peer review

**DOI:** 10.7554/eLife.00799

**Published:** 2013-04-30

**Authors:** Randy Schekman, Fiona Watt, Detlef Weigel

**Keywords:** scientific publishing, publishing, peer review, *eLife*

## Abstract

All editorial decisions at *eLife* are taken by working scientists in a process that emphasizes fairness, speed and transparency.

One of the founding principles of *eLife* has been to assign authority for the editorial decisions to scientists who are still active in the research community. Although the same is true of many journals, particularly those run by scientific societies, *eLife* has introduced a number of innovations into the peer review process to ensure that editorial judgments are made in a way that is both decisive and fair to authors, and also more transparent than at most journals. We believe that these innovations—combined with our commitment to open access and to fully exploiting the potential offered by digital media (Schekman et al., 2012)—will allow us to publish some of the best work in the life and biomedical sciences in a way that benefits both authors and readers.

When a paper is submitted to *eLife*, it is assigned to one of the senior editors on the journal. This editor then makes the initial decision on the paper, usually after consulting colleagues, in particular members of the larger Board of Reviewing Editors (BRE), which contains at least one expert in the areas of science that *eLife* aims to cover. As with most journals, authors are asked during the submission stage to identify the most appropriate editors to handle their manuscripts, and to propose suitable referees, and they are also given the opportunity to request that certain editors and referees are excluded from the process.

If the work is judged to represent a significant achievement using a broad set of criteria, a full submission is encouraged and a reviewing editor is appointed from the BRE or from the senior editors. This reviewing editor acts as a referee, selects one or more additional referees and oversees the peer review process. About 43% of initial submissions make it through this initial stage, and the median time from receipt to initial decision is about three days.

An important element of the peer review process at *eLife* is that referees are directed to limit their recommendations to changes that bear directly on the major conclusions of the work. As it states on our web site:

1. We will only request new work, such as experiments, analyses, or data collection, if the data are essential to support the major conclusions.
2. The authors must be able to do any new work in a reasonable time frame. If the conclusions are not adequately supported by the existing data, the submission should be rejected.
3. Any requests for new work must fall within the scope of the current submission and the technical expertise of the authors.

The new approach being taken by *eLife* really kicks in once all the reviews have been received: the reviewing editor initiates an online consultation session in which each referee can see who the other referees are and what they wrote about the manuscript. Over the course of several days, the referees exchange views on the merits of the work. If the consensus is that the manuscript should be rejected—and about 40% of the manuscripts that have reached this stage to date have been rejected—the reviews are usually conveyed to the authors in full.

If, on the other hand, the consensus is that the manuscript is, in principle, acceptable for publication in *eLife*, but requires additional experiments or analysis, the referees work with each other to identify the additional studies that are required for acceptance. The reviewing editor then drafts a decision letter that explains these essential revisions; we prefer that these core comments are summarized in at most 1000 words. If it is agreed during the consultation session that some of the referees’ concerns do not affect the validity or overall conclusions of the manuscript, then these concerns are not included in the decision letter. (Such concerns are sometimes conveyed to the authors, but in these cases it is made clear to the authors that it is not essential to address these concerns in the revised manuscript.) All decisions, either favourable or unfavourable, are reviewed and approved by the Editor-in-Chief or one of the two Deputy Editors, plus one other senior editor. The end result is that the authors have a clear directive from the editors—a roadmap that, if navigated successfully, will virtually guarantee acceptance. Although the consultation among the referees adds some time to the decision process, the median time from receiving the full submission to the decision after peer review is currently running at 27 days.

The end result is that the authors have a clear directive from the editors—a roadmap that, if navigated successfully, will virtually guarantee acceptance.

When the revised manuscript is received, the reviewing editor can usually make an executive decision on whether or not the authors have addressed the major concerns conveyed in the decision letter in a satisfactory manner. This is one of the reasons why the median time from receiving the full submission to acceptance is presently less than 80 days. Finally, subject to approval by the authors, the decision letter after peer review and the author response to this decision letter are included in the HTML version of the article as part of our efforts to make the peer review process more transparent.

The responses of referees, authors and editors to this approach have been almost universally enthusiastic. Referees, in particular, have found the consultation session to be engaging and an improvement on the conventional approach in which reviews are cast into an electronic void and the referee often remains oblivious to the fate of the paper, and rarely learns who the other referees were. And, surprisingly perhaps, several authors of papers that were not accepted after initial or full review have told us that, despite their disappointment, they found the whole process of peer review—and, in some cases, rebuttal—to be fair and, in general, fast. It will be clear to readers that the *eLife* approach to peer review requires a significant commitment to the journal from researchers who already have many calls on their time. The editors are therefore financially remunerated in recognition of the work that they are putting into the journal.

As with any new process, some concerns were raised about our consultation stage, particularly the identification of referees to one another. Would a junior investigator feel reluctant to challenge the views of an established expert? Could we assure the confidentiality of the process? And what would happen in the event that a consensus could not be reached? To date, these issues have not presented a problem. Young scholars who have served as referees have not been reluctant to express contrary views, referees have maintained confidentiality (where desired) and, remarkably, a consensus has always been reached (although the relevant senior editor can be called on to make a final decision in the case of deadlock).

As *eLife* grows, the capacity of our editors and our procedures will be challenged. It is already obvious, however, that our fresh approach to editorial decision making is working well. And with other journals making similar innovations—and here we acknowledge the pioneering work of the *British Medical Journal*, *EMBO Journal* and some of the BMC journals in this regard—we are clearly entering a new era of fairer and more efficient peer review.
